# Modulation of Dendritic-Epithelial Cell Responses against *Sphingomonas Paucimobilis* by Dietary Fibers

**DOI:** 10.1038/srep30277

**Published:** 2016-07-25

**Authors:** Miriam Bermudez-Brito, Marijke M Faas, Paul de Vos

**Affiliations:** 1 Top Institute Food and Nutrition, Wageningen, The Netherlands; 2 University of Groningen, University Medical Center Groningen, dept. Pathology and Medical Biology, Groningen, The Netherlands

## Abstract

Non-fermenting Gram-negative bacilli, such as *Sphingomonas paucimobilis* (*S.paucimobilis*), are among the most widespread causes of nosocomial infections. Up to now, no definitive guidelines exist for antimicrobial therapy for *S. paucimobilis* infections. As we have shown that some dietary fibers exhibit pronounced immune-regulatory properties, we hypothesized that specific immune active dietary fibers might modulate the responses against *S. paucimobilis*. We studied the immunomodulatory effects of dietary fibers against *S. paucimobilis* on cytokine release and maturation of human dendritic cells (DCs) in co-cultures of DCs and intestinal epithelial cells (IECs). *S. paucimobilis* infection resulted in increased release of pro-inflammatory cytokines and chemokines by DCs/IECs; these effects were strongly attenuated by specific dietary fibers. Chicory inulin, sugar beet pectin, and both starches had the strongest regulatory effects. IL-12 and TNF-α were drastically diminished upon exposure to chicory inulin and sugar beet pectin, or both starches. High-maize 260, was more effective in the reduction of chemokine release than the others fibers tested. In summary, chicory inulin, sugar beet pectin, High-maize 260, and Novelose 330 attenuate *S. paucimobilis*-induced cytokines. These results demonstrate that dietary fibers with a specific chemical composition can be used to manage immune responses against pathogens such as *S. paucimobilis*.

Non-fermenting Gram-negative bacilli can be a major problem in the clinical environment, being the common cause of nosocomial infections. Opportunistic pathogens take advantages of underlying conditions and diseases to cause infection. *Sphingomonas paucimobilis* (*S. paucimobilis*) is one of these emerging opportunistic pathogens^1^. Although it is an organism of low clinical virulence, infections caused by *S. paucimobilis* can lead to septic shock^2^. Moreover, *S. paucimobilis* has been associated with a variety of infections in healthy and immunocompromised patients, including bacteremia^3^, pneumonia^4^, catheter-related infections^5^, meningitis^6^, peritonitis^7^, osteomyelitis, septic arthritis^8^, postoperative endophthalmitis^9^, urinary tract infections, and biliary tract infections^10^.

*S. paucimobilis* is a strictly aerobic and non-spore forming Gram-negative bacillus^11^ that is widely distributed in nature, especially in water and soil^3^. It has also been recovered from diverse sources in the hospital environment, including hospital water system, respiratory therapy equipment, and laboratory instruments^12^. *S. paucimobilis* is unique in its possession of ubiquinone 10 as its major respiratory quinone, and of glycosphingolipids instead of lipopolysaccharide (LPS)^13^, in their cell envelopes. It can survive under severe circumstances, as it can utilize a wide range of naturally occurring compounds as well as some types of environmental contaminants^14^.

To date, no definitive guidelines exist for antimicrobial therapy for *S. paucimobilis* infections^15^. Due to its pathogenic potential, it is important to choose the correct antibiotic or to find an alternative method to eliminate the action of this pathogen. Our previous studies have shown the immune regulatory potential of some dietary fibers^16^,^17^. Therefore, we hypothesized that dietary fibers might also modulate the action against *S. paucimobilis*. We applied a transwell co-culture system involving intestinal epithelial cells (IECs) and human dendritic cells (DCs) that mimics the *in vivo* situation in the gut^18^. The effects of the dietary fibers GOS, chicory inulin, sugar beet pectin, wheat arabinoxylan, barley β-glucan, High-maize 260 and Novelose 330 was studied after challenging the co-culture system with *S. paucimobilis.*

## Material And Methods

### Substrates

We studied the effects of Vivinal GOS (FrieslandCampina, Wageningen, The Netherlands), chicory inulin (Royal Cosun, Roosendaal, The Netherlands), sugar beet pectin (Dupont, Wilmington, USA), wheat arabinoxylan, barley β-glucan (Megazyme, Wicklow, Ireland), High-maize 260 and Novelose 330 (Ingredion, Westchester, USA) on cytokine release and maturation of DCs in co-cultures of IECs.

### Isolation and identification of Sphingomonas paucimobilis

The bacterial isolate used in this study was collected from distilled water from the cell-culture incubator at the University Medical Center Groningen, and plated on trypticase soy agar (TSA). Isolates were purified by subculturing on TSA.

### Generation of an epithelial cell monolayer

Human colon carcinoma Caco-2 cells were grown in flasks (T-25/75 cm2) in DMEM (Gibco, Life technologies, Bleiswijk, The Netherlands) supplemented with 10% fetal calf serum (Hyclone, Thermo Scientific, Breda, The Netherlands), 2 mM L-glutamine, 1% nonessential aminoacids (Gibco), and 50 μg/mL Penicillin-Streptomycin Solution (ATCC, The Netherlands). Thereafter, Caco-2 cells were seeded in the upper chamber of a transwell filter (3 μm pore size, 6.5 mm diameter; Corning, NY) for 15–21 days. The cells were grown to confluence until the trans-epithelial resistance (TER) reached 300 Ωcm^2^.

### DCs

DCs, generated from umbilical cord blood CD34^+^ progenitor cells (haematopoietic stem cells) were supplied by MatTek Corporation (Ashland, MA)^19^. DCs were cultured according to the manufacturer’s instructions. Umbilical cord blood was obtained from a local hospital or from the National Disease Research Interchange (NDRI, Philadelphia, PA) following Institutional Board Approval (IRB).

### Co-cultures of Caco-2 cells and DCs

Briefly, IEC-containing filters were positioned upside down in a 6-well plate, and a drop containing 5 × 10^4^ DCs was placed onto the membrane. DCs were allowed to adhere for 4 h at 37 °C. Afterwards, the transwell inserts were turned again into the 24-well plates, and co-cultured with 2 × 10^5^ DCs/well at the lower chamber for 24 h. Then, the co-cultures were pretreated with *S. paucimobilis* (10^7^−10^9 ^CFU/ml) for 24 h. Afterwards, the fibers were dissolved in culture medium at a final concentration of 400 μg/ml and added from the apical surface (top chamber) for a period of 24 h (37 °C, 5% CO_2_). Supernatants were collected for cytokine analysis.

Negative control co-cultures were not exposed to any bacteria or dietary fibers. Endotoxin levels as tested by a Limulus amebocyte lysate assay of all used dietary fibers samples were always below 0.3 × 10−3 μg^−1^ which has no effect on the responsiveness of the cells to avoid that effects might be caused by endotoxin contamination such as LPS.

### Cytokine expression

After 24 h of incubation cytokine levels in the supernatant were measured using a MilliPlex premixed cytokine assay, according to the manufacturer’s instructions (Linco Research Inc, MO). This customized kit measures simultaneously or several of the following molecules; human IFN-γ, IL-12p40, IL-1β, IL-1Ra, IL-10, IL-4, IL-6, IL-8, MCP-1/CCL2, MIP-1α/CCL3, RANTES/CCL5, and TNF-α. Concentration series of cytokine standards were prepared for the appropriate concentration range, and coupled beads were diluted ten times, resuspended and added to a pre-wetted filter plate. After washing the plate twice, standards, negative controls and samples (all in duplicate) were transferred into the plate (50 μl per well), and the plate was sealed and incubated on a shaker at 4 °C overnight (16–18 h) in the dark. After incubation, the plate was washed three times, detection antibodies were resuspended and diluted ten times and 25 μl was added to each well. The plate was incubated on a shaker at room temperature (RT) for 1 h in the dark, and after washing three times, 50 μl of streptavidin-phycoerythrin was added to each well and the plate was incubated on a shaker at RT for 30 min in the dark. After washing the plate three times, 125 μl of assay buffer was added per well, the plate was incubated on a shaker for 5 minutes and fluorescence was measured using a Luminex 100 System, and Star Station software.

### Flow cytometry and antibodies

The following antibodies were used for flow cytometry staining: anti-human leukocyte antigen-DR fluorescein isothiocyanate conjugated (HLA-DR; FITC), anti-human CD86 phycoerythrin, cyanine dye conjugated (PE/Cy7), and anti-human CD83 allophycocyanin conjugated (APC), with matched isotype controls (all from Biolegend, San Diego, CA). DCs were stained as described previously by Bermudez-Brito *et al.*^16^ and later analyzed using the FACSCalibur Flow Cytometer platform (BD Bioscience, San Jose, CA) and Flowjo 7.6.5 software. For each analysis 20,000 counts, gated on a FSC versus SSC dot plot, based on viability, were recorded. CD83, CD86 or HLA-DR isotype controls were used to set the gate to 99% negative cells.

### Statistics

Significance levels were determined by Kruskal Wallis test. Post-hoc analysis was performed using Bonferroni Multiple Comparison Test. Analysis were performed using NCSS 2007 software (Kaysville, UT). Results are expressed as mean ± SD. A *P*-value < 0.05 was considered statistically significant and denoted with an asterisk (*). In addition, differences between DCs-IECs treated with *S. paucimobilis* and DCs-IECs stimulated with *S. paucimobilis* and dietary fibers were also evaluated. A *p* < 0.05 was considered significant and is indicated in the figures with a pound sign (#).

## Results

### Dietary fibers are potent reducers of pro-inflammatory cytokines IL-12 and TNF-α production in *S. paucimobilis* challenged human dendritic cells and intestinal epithelial cells

**Figure 1:**
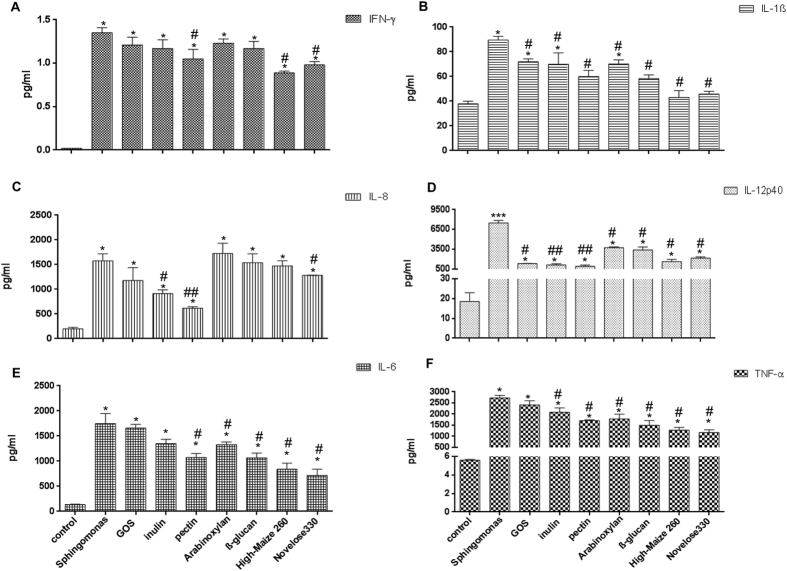
The production of IFN-γ, IL-1β (Panel A), IL-12(p40), TNF-α (Panel B), IL-6, and IL-8 (Panel C) after challenge with *Sphingomonas paucimobilis* in the presence and absence of dietary fibers. DCs and Caco-2 cells were pretreated with *S. paucimobilis* for 24 h, and then incubated another 24 h with dietary fibers. Culture supernatants were collected, and the cytokine levels were assessed by Luminex. The data shown are the means and SEM of four different experiments. Differences between *S. paucimobilis* stimulation compared to the controls (non-stimulated IECs/DCs) were considered statistically significant when the *p* values were less than 0.05 and were denoted with *. Differences between DCs-IECs treated with *S. paucimobilis* and DCs-IECs stimulated with *S. paucimobilis,* and dietary fibers were indicated with a pound sign (#).

IECs induce regulatory cytokine profiles in DCs and are under control of dietary fibers^16^,^17^. Here, we investigated how IECs, stimulated with different dietary fibers, and in the presence of *S. paucimobilis*, can impact DC activation across an epithelial barrier. We tested fibers with confirmed immunological activities; GOS, chicory inulin, sugar beet pectin, wheat arabinoxylan, barley β-glucan, High-maize 260, and Novelose 330. First, we studied the effects of these dietary fibers on the production of the pro-inflammatory cytokines IFN-γ, IL-12p40, TNF-α, IL-1β, IL-6, and IL-8 (Fig. 1) by DCs co-cultured with IECs challenged with *S. paucimobilis*.

As shown in Fig. 1A, the addition of the *S. paucimobilis* induced a sixty-fold increase in the production of IFN-γ (*p* < 0.05) in the IEC-DC co-culture system. Interestingly, expression of IFN-γ was been suppressed in the presence of specific dietary fibers such as sugar beet pectin, High-maize 260 or Novelose 330 (*p* < 0.05). A similar pattern was observed for IL-1β. The presence of *S. paucimobilis* induced a two-fold increase in IL-1β production (*p* < 0.05). This *S. paucimobilis*-induced IL-1β production was diminished upon fibers addition (*p* < 0.05). This effect was more pronounced when both starches were added (Fig. 1B).

As shown in Fig. 1D, *S.paucimobilis* is a potent inducer of the pro-inflammatory cytokine IL-12 (*p* < 0.005). *S.paucimobilis* induced a 300-fold increase in IL-12 production. Interestingly, IL-12 expression was drastically diminished by dietary fibers (*p* < 0.05), especially by chicory inulin or sugar beet pectin (*p* < 0.01). A similar pattern was observed for TNF-α. When IECs and DCs were challenged with *S. paucimobilis*, a 400-fold increase in TNF-α was observed (*p* < 0.05). The expression of this pro-inflammatory cytokine was significant decreased in the presence of the dietary fibers (*p* < 0.05), with exception of GOS. The resistant starches were the most potent TNF-α attenuating fibers (Fig. 1F).

The release of IL-8 may be induced by both TNF-α and IL-1β^20^. Thus, as expected, *S. paucimobilis* induced IL-8 release in human DCs and IECs (*p* < 0.05) (Fig. 1C). This enhancement was an eight-fold increase in IL-8 production. This effect was largely prevented by sugar beet pectin (*p* < 0.01), and to a lesser extent by chicory inulin and Novelose 330 (*p* < 0.05). GOS slightly decreased the release of IL-8 but this never reached statistical differences. Likewise, as shown in Fig. 1E, *S. paucimobilis* induced a twelve-fold increase in the production of IL-6 in the IEC-DC co-culture system (*p* < 0.05). Interestingly, the incubation of sugar beet pectin, wheat arabinoxylan, barley β-glucan, or both starches resulted in decreased release of this cytokine (*p* < 0.05), whereas GOS had no effect on IL-6 production. The addition of chicory inulin slightly decreased the production of IL-6 but this never reached statistical significant differences.

**Figure 2:**
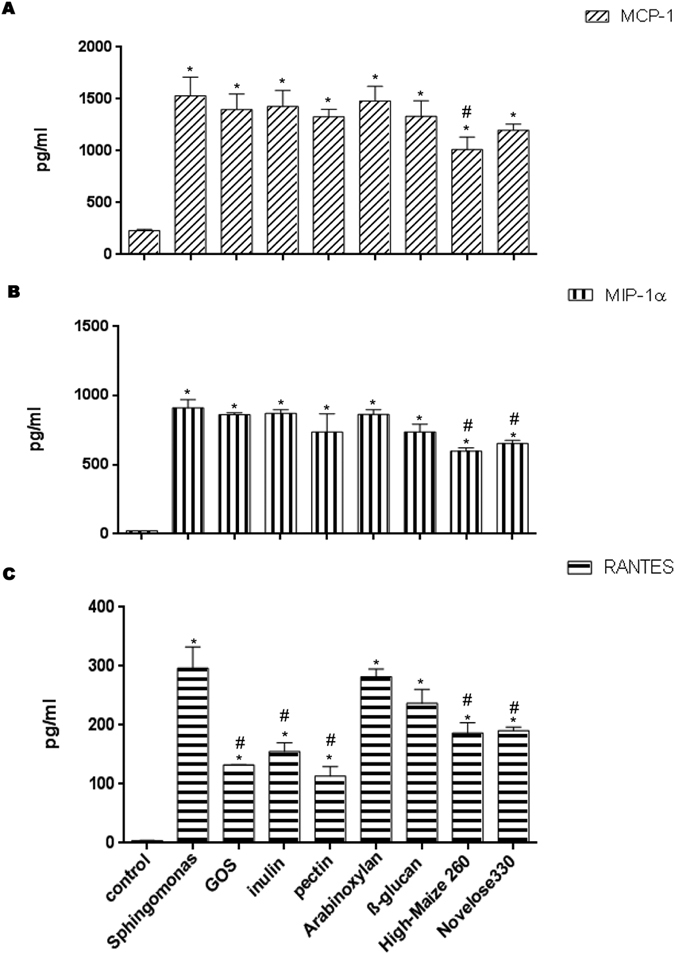
The production of the chemokines MCP-1/CCL2 (Panel A), MIP-1α/CCL3 (Panel B), and RANTES/CCL5 (Panel C) after challenge with *Sphingomonas paucimobilis* in the presence and absence of dietary fibers. DCs and Caco-2 cells were pretreated with *S. paucimobilis* for 24 h, and then incubated another 24 hours with dietary fibers. Culture supernatants were collected, and the cytokine levels were assessed by Luminex. The data shown are the means and SEM of four different experiments. Differences between *S. paucimobilis* stimulation compared to the controls (non-stimulated IECs/DCs) were considered statistically significant when the *p* values were less than 0.05 were denoted with *. Differences between DCs-IECs treated with *S. paucimobilis,* and DCs-IECs stimulated with *S. paucimobilis* and dietary fibers were indicated with a pound sign (#).

Further, we investigated whether the dietary fibers have an effect on the production of the chemokines MCP-1/CCL-2, MIP-1α/CCL3, and RANTES/CCL5 (Fig. 2) by DCs co-culture with IECs. Chemokines are secondary pro-inflammatory mediators that are induced by primary pro-inflammatory mediators such as IL-1β or TNF-α^21^.

As expected, *S. paucimobilis* induced a strong and substantial increase of MCP-1 compared to non-treated DCs/IECs (*p* < 0.05) (Fig. 2A). The only small but statistical significant change of the expression of MCP-1 was after High-maize 260 incubation (*p* < 0.05).

As shown in Fig. 2B, incubation with *S. paucimobilis* resulted in increased release of MIP-1α production (*p* < 0.05). Interestingly, this chemokine was drastically reduced by the addition of both starches, High-maize 260 or Novelose 330 (*p* < 0.05). Sugar beet pectin and barley β-glucan also reduced its production, but this never reached statistical significant differences. GOS, chicory inulin, and wheat arabinoxylan had virtually no effect on this chemokine.

Exposure to *S. paucimobilis* enhanced the production of RANTES (*p* < 0.05) (Fig. 2C). The levels of RANTES were notably decreased in the presence of the dietary fibers (*p* < 0.05), with exception of exposure to wheat arabinoxylan, and barley β-glucan. GOS and sugar beet pectin were the most potent inhibitors of *S. paucimobilis* induced RANTES production by the DCs.

### Dietary fibers induce a regulatory phenotype in the presence of *S. paucimobilis*

Next we investigated the effect of the dietary fibers on the regulatory cytokines IL-10, IL-1Ra, and IL-4 that counteract the effects of the proinflammatory cytokines^22^.

**Figure 3:**
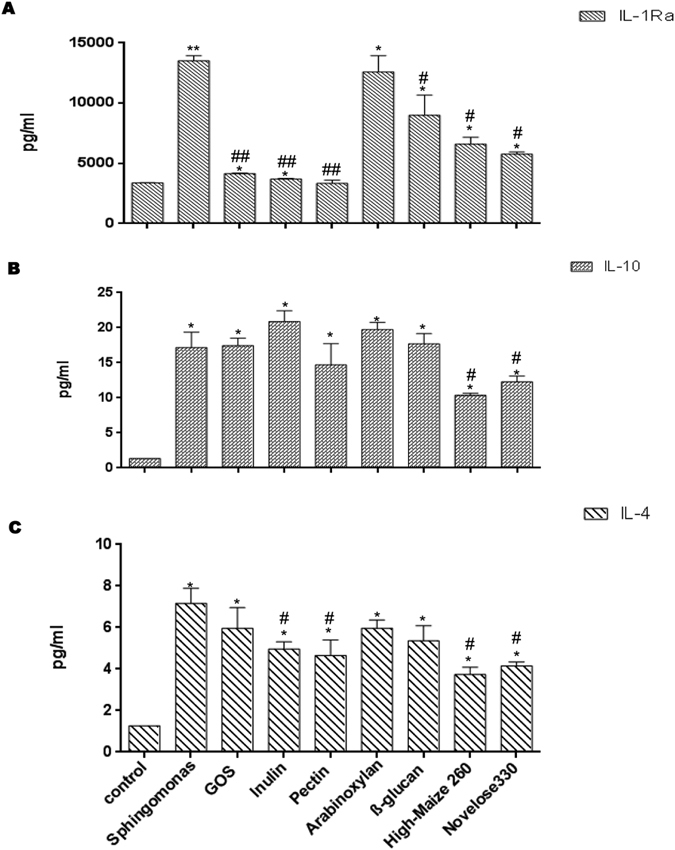
The production of IL-1Ra, IL-10, and IL-4 after challenge with *Sphingomonas paucimobilis* in the presence and absence of dietary fibers. DCs and Caco-2 cells were pretreated with *S. paucimobilis* for 24 h, and then incubated another 24 hours with dietary fibers. Culture supernatants were collected, and the cytokine levels were assessed by Luminex. The data shown are the means and SEM of four different experiments. Differences between *S. paucimobilis* stimulation compared to the controls (non-stimulated IECs/DCs) were considered statistically significant when the *p* values were less than 0.05, and were denoted with *. Differences between DCs-IECs treated with *S. paucimobilis,* and DCs-IECs stimulated with *S. paucimobilis* and dietary fibers were indicated with a pound sign (#).

As shown in Fig. 3A, incubation with *S. paucimobilis* resulted in a four-fold increased release of IL-1Ra production (*p* < 0.01). The production of IL-1Ra was decreased by all the dietary fibers tested (*p* < 0.05), with exception of wheat arabinoxylan. As shown in Fig. 3A, incubation with GOS, chicory inulin, or sugar beet pectin induced a similar production of IL-1Ra as that of non-stimulated DCs, and IECs (*p* < 0.01).

*S. paucimobilis* stimulation induced a twelve-fold increase in the production of IL-10 (*p* < 0.05) (Fig. 3B). This anti-inflammatory cytokine was increased in the presence of chicory inulin, wheat arabinoxylan, or barley β-glucan. However this never reached statistical significant differences, while High-maize 260, and Novelose 330 inhibited this cytokine (*p* < 0.05).

Incubation of the co-culture with *S. paucimobilis* induced a seven-fold increase in IL-4 production (*p* < 0.05) (Fig. 3C). Expression of IL-4 has been decreased in the presence of specific dietary fibers such as chicory inulin, sugar beet pectin, or both starches (*p* < 0.05) while GOS, wheat arabinoxylan, or barley β-glucan had no effect.

### Dietary fibers increased the IL-10/IL-12 ratio of *S. paucimobilis*-treated DCs/IECs

**Figure 4:**
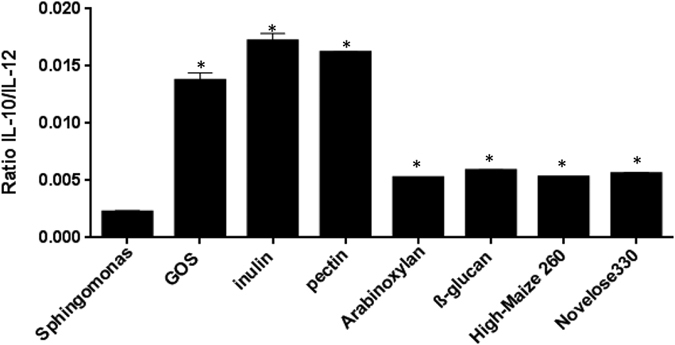
Ratio of IL-10/IL-12 upon incubation with *Sphingomonas paucimobilis* and the dietary fibers. Mean and SEM of the IL-10/IL-12 ratios is plotted for the different fibers as percentage of controls, which were set to 100%. *P*-values < 0.05 are denoted with an asterisk (*).

The IL-10/IL-12 ratios were calculated as a measure for the balance between anti-inflammatory and pro-inflammatory effects. Interestingly, the IL-10/IL-12 ratio was significant increased in the presence of the dietary fibers (Fig. 4). As shown in Fig. 4, the IL-10/IL-12 ratio was profoundly skewed in DCs more towards the anti-inflammatory IL-10 in the presence of GOS, chicory inulin, and sugar beet pectin (*p* < 0.05) while wheat arabinoxylan, barley β-glucan, and starches had a moderate but statistical significant enhancing effect on this ratio (*p* < 0.05).

### Dietary fibers reduced CD86 expression

To determine whether *S. paucimobilis* changes the phenotype of DC in the presence or absence of dietary fibers CD83, CD86, and HLA-DR expression was quantified in the DCs co-cultured with IECs.

As shown in Table 1, *S. paucimobilis* increased the expression of CD83, CD86, and HLA-DR on IECs-DCs co-culture. The attenuation of *S. paucimobilis*-induced pro-inflammatory phenotype by dietary fibers was substantiated by the FACS analysis where we observed a reduction of CD86 (*p* < 0.05) (Table 1). Interestingly, the starches High-maize 260 and Novelose 330 diminished its expression similar to non-treated DCs/IECs. CD83 expression was significant decreased by chicory inulin, sugar beet pectin, and wheat arabinoxylan (*p* < 0.05). As shown in Table 1, HLA-DR expression was increased by GOS, wheat arabinoxylan, and barley β-glucan (*p* < 0.05), whereas chicory inulin, sugar beet pectin, and both starches decreased its expression (*p* < 0.05). Interestingly, Novelose 330 had the most pronounced decreased effect of HLA-DR expression.

## Discussion

Our previous studies showed that dietary fibers exhibit strong immune regulatory properties^16^,^17^. The mechanisms by which fibers exert these immune modulating effects are by suppression of the production of pro-inflammatory cytokines, increasing IL-10-IL-12 ratio, upregulating the release of neutralizing cytokines, and contributing to shifts in T-cell polarization. Our results demonstrate that TLRs are essential in the dietary fiber induced regulatory effects. Here, we hypothesized that these regulatory immune active dietary fibers can be instrumental in regulating responses against opportunistic pathogens such as *S. paucimobilis.*

*S. paucimobilis* has been implicated in a variety of community-acquired and nosocomial infections including bacteraemia/septicaemia, cutaneous infections and diarrhoea, but also in invasive and severe infections, e.g. septic arthritis and osteomyelitis^1^. Thus, it is important to find an alternative to eliminate the action of this pathogen. To the best of our knowledge, this is the first study addressing the effects of dietary fibers on *S. paucimobilis* infection. Dietary fibers were effective in attenuating the extreme pro-inflammatory responses observed after *S. paucimobilis* infection.

Our data demonstrate and confirm that *S. paucimobilis* is a potent inducer of pro-inflammatory cytokines^1^, especially IL-12 and TNF-α, and chemokines. Several microbial lipid antigens (Ag) have been identified that are presented by Ag-presenting molecule CD1d and activate NKT cells including glycosylceramides from *Sphingomonas* species, phosphatidylinositol mannosides from *Mycobacterium tuberculosis*, and lipophosphoglycan from *Leishmania donovani*^23^,^24^,^25^,^26^. These glycoseramides from the cell wall of Sphingomonas serve as direct targets for human NKT cells activation, and are an alternative for LPS that *S. paucimobilis* lacks, for innate recognition of Gram negative LPS-negative bacteria26 and are probably responsible for the strong cytokine responses. CD1d induction is mediated by the pro-inflammatory cytokine TNF-α^27^. Moreover, Krziwon *et al.*^28^ demonstrated that glycosphingolipids isolated from *S. paucimobilis*, particularly tetraglycosylated GSL-4A was able to induce the release of TNF-α, IL-6, and IL-1β by human mononuclear cells (MNC), in line with our results. Furthermore, as the release of IL-8 is induced by both TNF-α and IL-1β^20^, *S. paucimobilis* also enhanced its production in human DCs and IECs.

Lipopolysaccharide (LPS) also elicits strong immune response in this transwell model^18^. This response to LPS is stronger than with *S. paucimobilis.* However, the reason to choose *S. paucimobilis* as model instead of LPS is because of the goal to prove that dietary fibers can be applied for infections that are difficult to treat. *S. paucimobilis* is an organism that is hard to eradicate with antibiotics and associated with too strong immune responses especially in immunocompromised patients^29^ for which no adequate therapy is available.

The magnitude of the changes induced by *S. paucimobilis* reveals a potent inflammatory response characterized mainly by IL-12 and TNF-α, and to a lesser extent by the other pro-inflammatory cytokines, and chemokines. This enhancement was drastically diminished by dietary fibers. Particularly interesting is the dramatic reduction of IL-12 by chicory inulin and sugar beet pectin by more than 6-fold, and TNF-α upon starches exposure by more than 2-fold. These effects of dietary fibers are accomplished by direct interaction of the fibers with so-called pattern recognition receptors (PRRs) on the immune cells, such as Toll-like receptors (TLRs) and Dectin-1^17^,^30^. In previous studies we proved that epithelial cells have profound anti-inflammatory effects when they have been exposed to the dietary fibers studied here^16^,^17^. These effects are myD88 and thus Toll-like receptor dependent. That this anti-inflammatory effect of fibers on pathogen-induced inflammation is demonstrated in the absence of microbiota was rather surprising. Up to now anti-inflammatory properties of fibers such as inulin, starch, and pectins^31^,^32^,^33^,^34^ were attributed to effects on the production of the anti-inflammatory short-fatty acids (SCFAs) acetate, propionate and butyrate by specific gut bacteria^35^. Here we show that these anti-inflammatory effects can also be accomplished by direct interaction of specific dietary fibers with mucosal cells. The most pronounced anti-inflammatory effects were observed upon exposure to chicory inulin, sugar beet pectin, or the resistant starches.

The beneficial effects of chicory inulin, sugar beet pectin, and the resistant starches are probably due to their specific chemical composition. Inulin is a plant storage polysaccharide that consists of linear chains of fructose residues with a β-(2-1) linkage and has many beneficial effects after binding to specific PRRs such as TLR2^30^. Pectin does not have these linkages but contains polysaccharides such as homogalacturonan and rhamnogalacturonan I and II, with side chains of arabinans, galactans, and arabinogalactans^36^. It is probably these groups that are responsible for the wide range of immunomodulatory activities reported for pectin^37^. High-maize 260 and Novelose 330, consist of 85 and 82 w/w solely glucose (98%), respectively. Both resistant starches are formed by ca. 83% total starch and 36% resistant starch^17^. These structural features seem to determine the effects of these fibers on the immune system^16^,^17^. These data support the growing evidence that high intake of specific dietary fibers is inversely associated with lower levels of pro-inflammatory markers in individuals with diabetes, hypertension, or obesity^38^, and a more regulatory status^39^. At the same time our data demonstrate that the current societal trend to non-specifically enhance dietary intake might be less effective as it are specific chemical structures that have to be hold responsible for the beneficial effects of dietary fibers.

One of the notable findings from our previous studies is that upon dietary fibers supplementation, a regulatory phenotype was observed. In the present study, the fibers tested tend to decrease the release of the pro-inflammatory cytokines and chemokines induced by *S. paucimobilis* by more than 1.5-fold, leading to values comparable to non-stimulated cells. Taken together, our data points towards a regulatory status promoted by dietary fibers *per se* through direct effects on mucosal cells, supporting the idea that there is a crosstalk between IECs, DCs and dietary fibers which induces a regulatory immune environment^16^, even when pathogens are present.

Co-stimulatory molecules are a class of receptors that play a crucial role in the innate immune response^40^. Mature DCs can prime naïve T cells and initiate primary T cells immune response^41^. Previously, we have shown that the currently applied dietary fibers reduced the maturation surface markers CD83 and CD86 on DCs^16^,^17^. This was one of the reasons to investigate potential links between dietary fibers and *S. paucimobilis* and the expression of surface markers, such as CD83, CD86 and HLA-DR. All the fibers decreased CD86 expression on DCs. This downregulation can be one of the mechanism by which dietary fibers decrease the production of pro-inflammatory cytokines by immune cells as it has been shown that CD80/CD86 via NF-kB induces several cytokines such as IL-6^42^. Interestingly, the fibers with the most pronounced anti-inflammatory effect i.e. chicory inulin, sugar beet pectin, and starches, decreased HLA-DR, a biomarker of inflammation^43^ suggesting a downregulation of antigen presentation. Furthermore, chicory inulin and sugar beet pectin also decreased CD83.

Another possible mechanism by which dietary fibers induce anti-inflammatory responses is by changing the balance in IL-10/IL-12 ratio. The IL-10/IL-12 ratio has been effectively used as a measure of the balance between anti- and pro-inflammatory state, or Th1 versus Th2 dominance^44^,^45^. It can be concluded on the basis of our data that dietary fibers skew this ratio in DCs more towards IL-10, and thus induce a more regulatory balance. Interestingly, the most pronounced anti-inflammatory effects were observed upon exposure to chicory inulin and sugar beet pectin.

## Conclussions

To the best of our knowledge, this is the first study addressing the effects of dietary fibers on *Sphingomonas*. Our data suggest that specific dietary fibers are efficacious in reducing pro-inflammatory cytokines release upon *S. paucimobilis* infections. Using dietary fibers to manage immune responses against pathogens might be an efficacious way to reduce the use of antibiotics.

## Additional Information

**How to cite this article**: Bermudez-Brito, M. *et al.* Modulation of Dendritic-Epithelial Cell Responses against *Sphingomonas Paucimobilis* by Dietary Fibers. *Sci. Rep.* **6**, 30277; doi: 10.1038/srep30277 (2016).

## Figures and Tables

**Table 1:** Expression of the activation markers CD83, CD86, and HLA-DR on the DCs, stimulated with *Sphingomas paucimobilis* and the dietary fibers GOS, chicory inulin, sugar beet pectin, wheat arabinoxylan, barley β-glucan, High-maize 260, or Novelose 330.

	Control	*Sphingomonas paucimobilis*	GOS	Chicory inulin	Sugar beet pectin	Wheat arabinoxylan	Barley β-glucan	High-maize 260	Novelose 330
CD83	12,7 ± 0,12	16,07 ± 0,58*	12,8 ± 0,78	10,65 ± 0,20*^,#^	9,88 ± 0,63*^,#^	11,19 ±0,84^#^	13,2 ± 1,23	13,6 ± 0,53	13,63 ± 0,58
CD86	11,35 ± 0,05	13,9 ± 0,08*	8,12 ± 0,61*^,#^	8,81 ± 0,40*^,#^	7,07 ± 0,63*^,#^	7,1 ± 0,17*^,#^	7,11 ± 0,21*^,#^	11,16 ± 0,55^#^	11,2 ± 0,74^#^
HLA-DR	60,8 ± 5,2	71,0 ± 0,16*	79,93 ± 1,88*^,#^	47,15 ± 0,13*^,#^	51,75 ± 1,27*^,#^	77,5 ± 0,53*^,#^	77,06 ± 2,52*^,#^	67,6 ± 2,8^#^	44,1 ± 1,88*^,#^

Numbers indicate the percentage of positive cells in the gate and SD of three different experiments. Differences between *S. paucimobilis* stimulation compared to the controls (non-stimulated IECs/DCs) were considered statistically significant when the *p* values were less than 0,05, and were denoted with *. Differences between DCs-IECs treated with *S. paucimobilis* and DCs-IECs stimulated with *S. paucimobilis* and dietary fibers are indicated with a pound sign (#).
